# Purinergic Signaling and Aminoglycoside Ototoxicity: The Opposing Roles of P1 (Adenosine) and P2 (ATP) Receptors on Cochlear Hair Cell Survival

**DOI:** 10.3389/fncel.2019.00207

**Published:** 2019-05-15

**Authors:** Shelly C. Y. Lin, Peter R. Thorne, Gary D. Housley, Srdjan M. Vlajkovic

**Affiliations:** ^1^Department of Physiology and The Eisdell Moore Centre, Faculty of Medical and Health Sciences, The University of Auckland, Auckland, New Zealand; ^2^Department of Physiology and Translational Neuroscience Facility, School of Medical Sciences, University of New South Wales, Sydney, NSW, Australia

**Keywords:** aminoglycoside ototoxicity, cochlear explant, sensory hair cells, adenosine, ATP, P1 receptor, P2 receptor, purinergic signaling

## Abstract

Purinergic signaling regulates important physiological processes and the homeostatic response to stress in the cochlea via extracellular nucleosides (adenosine) and nucleotides (ATP, UTP). Using a previously established organotypic culture model, the current study investigated the effect of purinergic P1 (adenosine) and P2 (ATP) receptor activation on the survival of the sensory hair cell population in the cochlea exposed to the ototoxic aminoglycoside neomycin. Organ of Corti explants were obtained from C57BL/6 mice at postnatal day 3 (P3) and maintained in normal culture medium (with or without purine receptor agonists or analogs) for 19.5 h prior to neomycin exposure (1 mM, 3 h) followed by a further incubation for 19.5 h in culture medium. The cochlear explants were then fixed in 4% paraformaldehyde (PFA) and sensory hair cells labeled with Alexa 488-phalloidin. Neomycin induced a substantial loss of the sensory hair cells, mostly in the middle segment of the cochlea. This neomycin-induced ototoxicity was unaffected by the addition of P2 receptor agonists (ATP and UTP) in the culture medium, whilst the addition of their slowly-hydrolyzable analogs (ATPγS, UTPγS) aggravated neomycin-induced sensory hair cell loss. In contrast, the activation of P1 receptors by adenosine or adenosine amine congener (ADAC) conferred partial protection from neomycin ototoxicity. This study demonstrates a pro-survival effect of P1 receptor stimulation, whilst prolonged activation of P2 receptors has an opposite effect. Based on these findings, we postulate that P1 and P2 receptors orchestrate differential responses to cochlear injury and that the balance of these receptors is important for maintaining cochlear homeostasis following ototoxic injury.

## Introduction

Aminoglycoside antibiotics are used in treatment of tuberculosis and serious Gram-negative bacterial infections such as bacterial endocarditis, urinary tract infections, and pneumonia (Rybak and Ramkumar, 2007). A common side-effect associated with aminoglycoside use in humans is damage to the sensory hair cells of the inner ear leading to their death and a significant loss of hearing and vestibular function (Schacht et al., 2012). Cochlear damage is generally observed with the use of amikacin, kanamycin and neomycin, whereas the use of streptomycin and gentamicin is associated with vestibular toxicity (Forge and Schacht, 2000). Neomycin is commonly used to study ototoxicity and its mechanisms *ex vivo* (Löwenheim et al., 1999; Wang et al., 2003; Vintila et al., 2009). The transduction channels (MET channels) located at the tips of the stereocilia contribute to uptake of aminoglycosides into the sensory hair cells (Marcotti et al., 2005; Alharazneh et al., 2011). Inside the hair cells, the formation of an aminoglycoside-iron complex can react with electron donors, such as arachidonic acid, to form reactive oxygen species (ROS) (Poirrier et al., 2010). The superoxide, hydroxyl radical, and hydrogen peroxide in turn activate stress-activated protein kinase JNK, which translocates to the nucleus to activate downstream genes involved in the cell death pathways (Poirrier et al., 2010; Karasawa and Steyger, 2011). The excessive ROS production can exhaust endogenous antioxidant capacity, increase intracellular calcium levels, cause mitochondrial calcium overload, with release of cytochrome c and subsequently caspase-dependent cell death (Pinton et al., 2008; Karasawa and Steyger, 2011). As the mammalian auditory hair cells do not regenerate, their loss and the resulting hearing loss are permanent.

We and others have shown that purine and pyrimidine nucleotides acting via P2 receptors regulate aspects of normal cochlear function and its response to stress (Thorne et al., 2004; Lee and Marcus, 2008; Housley et al., 2009, 2013). P2R are activated by tri- and di-phosphate nucleosides, such as ATP, UTP, ADP, and UDP. There are two families of P2 receptors, ATP-gated ion channels (assembled from P2X receptor subunits 1–7) and the metabotropic G protein-coupled P2Y receptors (subtypes 1, 2, 4, 6, 11, 12, 13, 14). The majority of P2 receptors are found in the mammalian cochlea and are differentially distributed in cochlear tissues. P2 receptors distributed in the sensory and non-sensory epithelium of the organ of Corti and in the auditory nerve have distinctive roles in sensory transduction, auditory neurotransmission and the maintenance of cochlear homeostasis (Housley et al., 2006, 2009; Lee and Marcus, 2008). High densities of P2 receptors are present on the cuticular plates and stereocilia of the hair cells and their synapses with the primary afferent neurons located in the spiral ganglion (Housley et al., 1999; Szücs et al., 2004; Huang et al., 2010). Whilst the P2Y receptors (subtypes 1, 2, and 4) may be involved in ATP-induced Ca^2+^ release in the outer hair cells (OHCs) (Mammano et al., 1999), P2X_2_ receptors located in the apical region of the sensory hair cells likely have a role in cell depolarization, regulation of K^+^ extracellular concentration and cochlear micromechanics (Housley et al., 2009, 2013). ATP release from cochlear tissues can be triggered by acoustic overstimulation (Muñoz et al., 2001), and ATP release sites have been identified in the organ of Corti (Wangemann, 1996) and marginal cells of the stria vascularis (White et al., 1995). Connexin and pannexin hemichannels are likely the principal conduits for ATP release from non-sensory cells within the cochlea (Tang et al., 2008; Zhao, 2016). Regarding the role of ATP in cochlear stress responses, ATP released during acoustic overstimulation acts on P2X_2_ receptors in the cochlear partition to produce a shunt conductance for K^+^ and thus reduce the driving force for sound transduction (Thorne et al., 2004). This ATP-mediated shunt conductance for K^+^ represents a regulatory mechanism that provides homeostatic control of cochlear sensitivity and is associated with protection of hair cells from acoustic trauma (Housley et al., 2013). Others have shown that ATP signaling is important for cell-to-cell communication and essential for sensing hair cell damage in the cochlea and initiating cell death and repair pathways (Gale et al., 2004).

Endogenous ATP levels in cochlear fluids are tightly regulated by ectonucleotidases, a heterologous group of surface located enzymes hydrolyzing extracellular nucleotides to their respective nucleosides (Vlajkovic et al., 1996, 1998), and their expression has been demonstrated in the mouse and rat cochlea (Vlajkovic et al., 2002, 2006; O’Keeffe et al., 2010). Ectonucleotidases have a role to limit the spatio-temporal activity of extracellular nucleotides (Vlajkovic et al., 2009) and these enzymes are an integral component of the purinergic signaling system in the cochlea as they regulate the balance between P2 (ATP) and P1 (adenosine) receptors. P1 receptors are the family of G protein-coupled receptors (A_1_, A_2A_, A_2B_, A3) activated by adenosine. The extensive receptor-specific distribution of these receptors in the rat cochlea suggests a complex role for adenosine in regulation of cochlear function and response to stress (Vlajkovic et al., 2007). The adenosine A_1_R is of particular interest in cochlear survival in adverse conditions, as the activation of this receptor reduces oxidative stress, limits glutamate excitotoxicity, and inhibits voltage-gated Ca^2+^ channels, which can prevent downstream activation of apoptotic and necrotic cell death pathway (Vlajkovic et al., 2009). The otoprotective effect of the A_1_R agonists has been demonstrated after acoustic trauma (Vlajkovic et al., 2010, 2014; Wong et al., 2010) and with cisplatin ototoxicity (Whitworth et al., 2004; Gunewardene et al., 2013).

This background of purinergic involvement in cochlear response to stress led us to investigate the effect of P2 receptor agonists (ATP, UTP) and their slowly hydrolyzable analogs (ATPγS and UTPγS) on cochlear hair cell survival during exposure to the ototoxic antibiotic neomycin. Here we also investigated the effect of adenosine receptor (AR) activation on cochlear responses to neomycin to determine the overall effect of purinergic signaling on the maintenance of the sensory cell population in ototoxic conditions.

## Materials and Methods

### Animals

C57BL/6 mice at postnatal day 3 (P3) were used in this study. All experimental procedures were approved by The University of Auckland Animal Ethics Committee.

### Organ of Corti Tissue Culture

The organ of Corti from C57BL/6 mouse pups (P3) was micro-dissected and maintained on collagen coated coverslip in Dulbecco’s modified Eagle medium (DMEM, Thermo Fisher) using previously described protocol (Lin et al., 2018). Following micro-dissection, the organ of Corti explants were incubated at 37°C with 5% CO_2_ for a total of 42 h. The incubation timeframe included a pre-incubation in normal culture medium for 19.5 h, an ototoxic injury by neomycin (Sigma Aldrich, 1 mM) for 3 h and a post-incubation of 19.5 h in normal culture medium. This timeframe (and neomycin concentration) was established by titrating the concentration and timing to induce approximately 50% of hair cell loss, which allows a change in either direction to be observed. The pre-incubation is required to stabilize tissue preparation. The post-incubation improves the consistency of hair cell loss by providing a timeframe for sensory cell death and repair of the reticular lamina.

### Supplementation of P1 and P2 Receptor Agonists

To test the effect of P1 and P2 receptor agonists on neomycin ototoxicity, each agonist was added separately to the normal culture medium and incubated with cochlear explants for a total of 42 h. ATP and UTP were supplemented to the culture media at 100 μM as they are quickly hydrolyzed by ectonucleotidases. The slowly-hydrolyzable analogs of ATP and UTP (ATPγS and UTPγS) were supplemented at 1 uM due to their resistance to enzymatic hydrolysis. The P1R agonists used in this study included adenosine (100 μM), which activates all AR, and adenosine amine congener (ADAC; 1 μM), that predominantly activates the A_1_ adenosine receptor (A_1_R) at this concentration. These concentrations were based on assumption that adenosine is quickly removed from the extracellular space by nucleoside transporters, whilst ADAC remains in the extracellular space. Sensory hair cells were labeled with phalloidin, followed by epifluorescence imaging and quantitative analysis of sensory hair cells. To investigate the effect of P1 and P2 receptors on cochlear injury response, neomycin (1 mM) was added to the nucleotide-enriched medium in the middle of the timeframe for 3 h.

### Tissue Processing and Imaging

Cochlear explants were fixed with 4% PFA (30 min at room temperature) following the 42-h incubation in culture medium, washed with 0.1M PBS and permeabilised with 1% Triton X-100 in 0.1M PBS for 1 h. Sensory hair cells were labeled with Alexa 488-phalloidin (1:600) and a series of images were captured along the entire length of the cochlea using an Eclipse 80i Nikon microscope (Japan) at high resolution (2560 × 1920 pixels). The entire sensory epithelium (apex to hook) were reconstructed from the images using Adobe Photoshop ver. 19.1.3.

### Hair Cell Counting

Hair cells were counted along the entire cochlear coil. The organ of Corti was divided into 20 regions of equal length (5% each) and the results were presented as cochleograms. The proportion of missing or surviving hair cells was determined for each region in the following way. Because of the disordered state of the neomycin-treated organ of Corti, the number of surviving cells was determined by counting the number of cells with discrete stereociliar bundles (regardless of condition) using markers in the Cell Count plug-in of the ImageJ software (v. 1.46r, NIH, United States). The structural appearances of the stereocilia were noted as qualitative observations. Finally, a cochleogram showing percentage of surviving IHC and OHC was plotted as described previously (Viberg and Canlon, 2004).

### Neomycin Uptake in Cochlear Hair Cells

Neomycin-Texas Red conjugate (NTR) was used to study the uptake of neomycin in cochlear hair cells. NTR is a fluorescently-tagged neomycin produced by conjugating neomycin sulfate and succinimidyl ester of the Texas red dye. To make the NTR stock solution (100 mM), 0.095 g of neomycin (Sigma Aldrich) was dissolved in 70% v/v of milliQ water with 17.6% v/v of 0.5 M potassium carbonate and 12% v/v of 2.5 μM Texas Red sulfonyl chloride (Invitrogen). The mixture was incubated at 4°C, protected from light, for 3 days. NTR stocks were aliquoted and stored at -20°C. In this study, the NTR uptake was measured in the presence or absence of a slowly hydrolyzable P2R agonist ATPγS, as the P2R agonist may increase neomycin uptake by the hair cells. Sensory hair cell integrity was fundamental to this study since the loss of cell membrane integrity can cause leakage of NTR, hence the pre- and post-incubation period in normal culture medium was omitted. Following the dissection of the sensory epithelium, the tissue explants were incubated for 3 h with NTR (1 mM) in normal culture medium supplemented with ATPγS (1 μM). At the end of the 3-h incubation, cochlear explants were fixed with 4% PFA for 30 min, washed with 0.1 M PBS, permeabilised for 1 h with 1% Triton X-100, and labeled with Alexa Fluor 488-Phalloidin (1:600, 1 h at RT). After washing with PBS, the tissues were mounted using an antifade mounting medium (Citiflour, Leicester, United Kingdom). Phalloidin labeling and NTR uptake were imaged using an epifluorescence microscope (Eclipse 80i, Nikon). NTR pixel intensity was quantified using the ImageJ software to estimate neomycin uptake into the hair cells. The areas of IHC and OHC were selected as separate regions of interest (ROI) and the mean pixel intensity measured across all surviving cells. Background pixel intensity in a non-labeling region of the cochlea was deducted from the pixel intensity of the ROI to control for inter-cochlear variations.

### Adenosine A_1_R Distribution in the Cochlea

Adenosine A_1_ receptor distribution in the mouse (C57BL/6) cochlea was determined by immunolabeling of cochlear cross sections and surface preparations. These techniques are briefly described below.

#### Cochlear Cryosections

The cochleae were extracted from the temporal bones at P3, superfused with 4% PFA through the round window and fixed in 4% PFA overnight (4°C). On the following day, the cochleae were washed with 0.1M PBS and decalcified in Shandon TBD-1 Decalcifier (Thermo Fisher) for 30 min. Following wash in PBS, the cochleae were cryoprotected overnight with 30% sucrose in 0.1 M PBS at 4°C. The cochleae were then mounted in Tissue-Plus Optimal Cutting Temperature Compound (OCT, Fisher Scientific), snap-frozen with *n*-pentane and stored at -80°C. Cochleae were cryosectioned at 30 μm and immunostained with A_1_R-specific antibody using a floating section technique.

#### Cochlear Surface Preparations

Following decapsulation and micro-dissection of the organ of Corti, the cochlear explants were incubated in normal culture medium as described in Section “Organ of Corti Tissue Culture.” After 42 h, the cochlear explants were fixed with 4% PFA for 30 min at RT, washed in 0.1 M PBS (3 × 10 min) and immunostained with A_1_R-specific antibody.

#### Immunohistochemistry and Imaging

Cochlear cryosections and surface preparations were permeabilised with 1% Triton X-100 in 0.1 M PBS and blocked using 10% normal donkey serum for 1 h at RT, followed by incubation with the primary A_1_R antibody (rabbit anti-mouse, Alomone Labs, Jerusalem, Israel, 1:100 dilution) overnight at 4°C. In control tissues, the primary antibody was omitted or pre-absorbed for 3 h with the blocking peptide (1:1, Alomone Labs, Jerusalem, Israel). The next day, the wholemounts and cryosections were washed (3 × 10 min) with 0.1 M PBS and incubated with the secondary antibody (Alexa-488 donkey anti-rabbit, 1:400; Life Technologies) for 2 h at RT. Cochlear tissues were then mounted in Citifluor for imaging.

The surface preparations of the organ of Corti were double labeled with Alexa-647 Phalloidin (1:600 dilution; 1 h at RT) to visualize sensory hair cells. The A_1_R immunofluorescence and phalloidin labeling were imaged simultaneously under a laser scanning confocal microscope (FluoViewTM FV1000, Olympus) and processed with Olympus FluoView ver.1.7 software. For the surface preparation, a series of six to eight optical sections (Z-stacks) were collected from the tip of the stereocilia at every 2 μm. The images were obtained from two animals (four cochleae) followed by qualitative image analysis.

### Statistical Analysis

Three arbitrary segments corresponding to the apical, middle, and basal region of the cochlea were defined for statistical analysis of hair cell survival. The apical segment included the apical 30% of the organ of Corti, the middle segment included the middle 40% and the basal segment the last 30% of the organ of Corti. Data are presented as mean ± SEM. One-way ANOVA was used for comparisons of multiple groups (i.e., different treatments) followed by Tukey’s multiple comparison test. To examine the effect of two categorical variables (treatments and cochlear segments) on hair cell survival, two-way ANOVA was used followed by the same *post hoc* analysis. The α level was set at 0.05.

## Results

### Neomycin-Induced Hair Cell Loss

**Figure 1:**
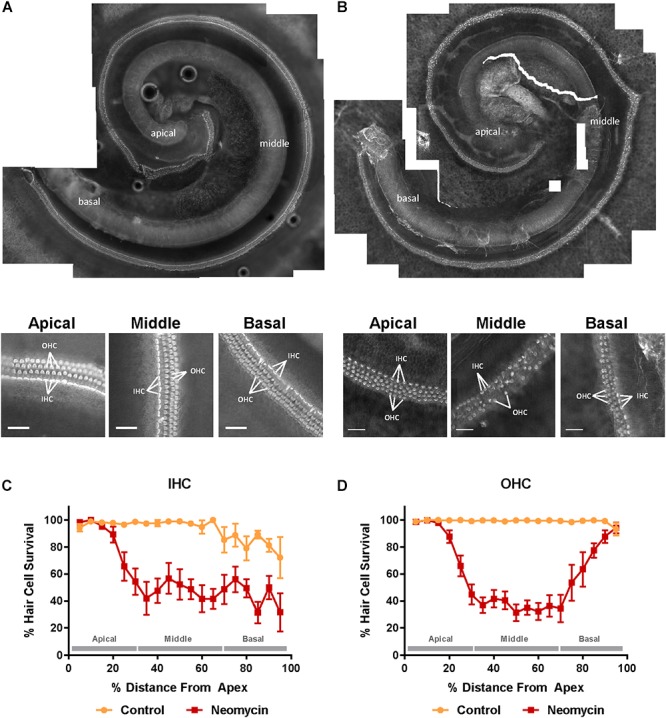
Effect of neomycin on hair cell survival in the mouse P3 organ of Corti explant detected with Alexa Fluor 488-Phalloidin fluorescence imaging. **(A)** Intact cochlear explant incubated for 42 h in normal culture medium. Higher resolution images below show excellent preservation of sensory hair cells in the apical, middle, and basal segment. **(B)** The P3 mouse organ of Corti explant exposed to neomycin (1 mM) for 3 h in the middle of the 42 h timeframe. Inner and outer hair cell (IHC, OHC) preservation was generally very good in the apical region of the cochlea, with a gradient to reduced preservation in the middle segment and poor among IHC with some loss of OHC in the basal segment. Scale bar = 30 μm**. (C)** IHC were equally susceptible to neomycin toxicity in the middle and the basal segment of the cochlea resulting in an L-shaped cochleogram. **(D)** In contrast, OHC were most susceptible to neomycin in the middle segment of the cochlea resulting in a U-shaped cochleogram. Data presented as mean ± SEM. Control explants, *n* = 7; explants exposed to neomycin, *n* = 8.

The structural integrity of the organ of Corti incubated in normal culture medium for 42 h was excellent (Figure 1A). As expected, exposing the organ of Corti to neomycin (1 mM, 3 h) in the middle of the timeframe adversely affected the survival of the sensory hair cells in the neonatal organ of Corti (Figure 1B). Hair cell counts, presented as cochleograms (number of present cells vs. distance along the cochlea), demonstrated a spatial gradient of sensitivity to neomycin ototoxicity. Inner hair cells (IHCs) exposed to neomycin demonstrated significantly lower survival rates in the middle (47.1 ± 8.8%, *p* = 0.023) and the basal (45.6 ± 6.6%, *p* = 0.017) segment compared to the apical segment (81.0 ± 8.8%, Figure 1C), resulting in ‘L-shaped’ cochleogram. In contrast, quantitative analysis of the OHC survival following neomycin treatment demonstrated a ‘U-shaped’ cochleogram with a better preservation of OHC in the apical (79.0 ± 8.9%, *p* = 0.005) and the basal (72.0 ± 8.6%, *p* = 0.008) segments, compared to the middle segment (36.0 ± 6.0%, Figure 1D).

### Effect of P2 Receptor Stimulation on Cochlear Hair Cell Survival

**Figure 2:**
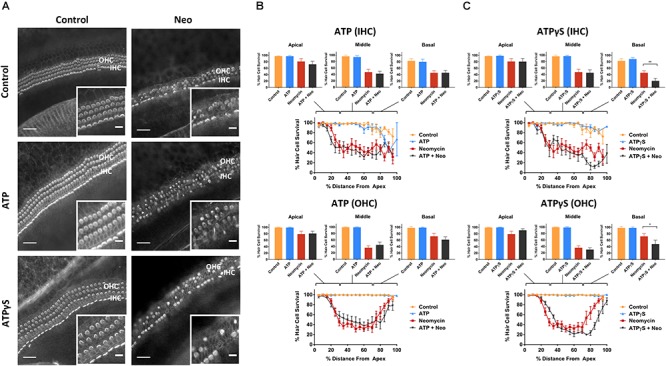
Effect of ATP or ATPγS supplementation on hair cell survival in the neomycin-treated mouse P3 organ of Corti explant. **(A)** Cochlear explants (basal segment) incubated in normal culture medium enriched with ATP or ATPγS for 42 h with or without neomycin administration (1 mM, 3 h) in the middle of the timeframe. IHCs, inner hair cells; OHCs, outer hair cells. Scale bars: main, 30 μm; inset, 10 μm. **(B)** ATP (100 μM) had no effect on the survival of IHC and OHC in normal culture medium or culture medium containing neomycin. Data presented as mean ± SEM (ATP, *n* = 7 and ATP + neomycin, *n* = 10). **(C)** In contrast, ATPγS (1 μM) significantly reduced the survival of both IHC (^∗∗^*p* < 0.01, one-way ANOVA) and OHC (^∗^*p* < 0.05) in the basal turn of the neomycin-treated explant. Data presented as mean ± SEM (ATPγS, *n* = 6 and ATPγS + neomycin, *n* = 6).

Control explants incubated for 42 h in normal culture medium supplemented with extracellular nucleotides (ATP, UTP, 100 μM) or their slowly hydrolyzable analogs (ATPγS, UTPγS, 1 μM), demonstrated excellent preservation of IHC and OHC and retention of organ of Corti structure (Figure 2A, 3A). Hair cell counts confirmed high survival rates across the sensory epithelium in explants incubated with extracellular nucleotides, similar to the survival rate in normal culture medium. This suggests that the activation of P2R under normal culture conditions has no adverse effects on the hair cells.

**Figure 3:**
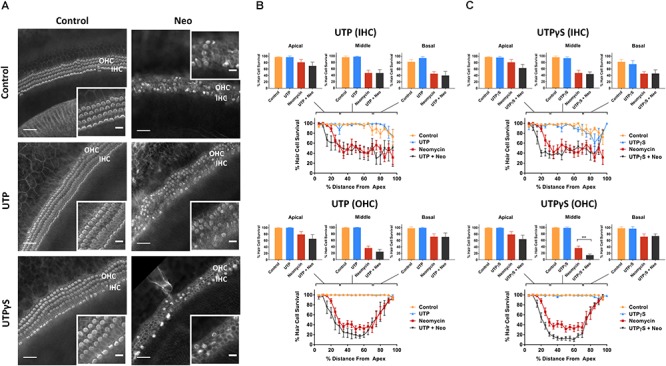
Effect of UTP or UTPγS supplementation on hair cell survival in the neomycin-treated mouse P3 organ of Corti explant. **(A)** Cochlear explants (middle segment) incubated in normal culture medium enriched with UTP or UTPγS for 42 h with or without neomycin administration (1 mM, 3 h) in the middle of the timeframe. IHCs, inner hair cells; OHCs, outer hair cells. Scale bars: main, 30 μm; inset, 10 μm. **(B)** UTP (100 μM) had no effect on the survival of IHC and OHC in normal culture medium or culture medium containing neomycin. Data presented as mean ± SEM (UTP, *n* = 6 and UTP + neomycin, *n* = 6). **(C)** UTPγS did not affect the survival of IHC, but significantly reduced the survival of OHC (^∗∗∗^*p* < 0.001, one-way ANOVA) in the middle segment of the cochlea. Data presented as mean ± SEM (UTPγS, *n* = 6 and UTPγS + neomycin, *n* = 7).

Exposing cochlear explants cultured in medium enriched with ATP or UTP to neomycin (1 mM) in the middle of the 42 h timeframe did not improve hair cell survival (Figure 2B, 3B). However, addition of ATPγS or UTPγS in the culture medium resulted in a significant segment-specific augmentation of hair cell loss, suggesting that prolonged activation of P2 receptors aggravates neomycin-induced injury (Figure 2C, 3C). ATPγS supplementation significantly (Figure 2C) increased neomycin ototoxicity in both IHC (from 45.6 ± 6.6 to 20.4 ± 8.1%, *p* = 0.0029, one-way ANOVA) and OHC (from 72.0 ± 8.6 to 48.0 ± 12.2%, *p* = 0.024) in the basal segment. Notably, ATPγS had no significant effect on the survival of the IHC and OHC exposed to neomycin in the apical and middle segments of the cochlea. In contrast, UTPγS supplementation, did not affect IHC survival, but significantly reduced OHC survival from 36.0 ± 6.0 to 13.8 ± 3.7% in the middle segment (*p* = 0.0058, one-way ANOVA, Figure 3C).

### Changes in NTR Uptake After ATPγS Treatment

**Figure 4:**
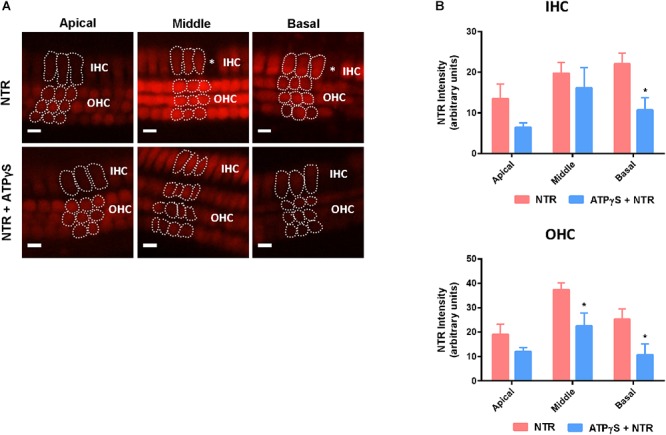
Changes in Neomycin-Texas Red (NTR, 1 mM, 3 h) uptake in the mouse P3 organ of Corti explant after ATPγS supplementation. **(A)** NTR labeling in the apical, middle, and basal segments of the cochlea with or without inclusion of ATPγS. Asterisks, missing hair cells. IHCs, inner hair cells; OHCs, outer hair cells. Scale bars: 10 μm. **(B)** NTR pixel intensity quantification. ATPγS (1 μM) had no effect on NTR uptake in the apical hair cells, but significantly reduced NTR uptake in the basal segment IHC and middle and basal segment OHC. Data presented as mean ± SEM; NTR, *n* = 10; NTR + ATPγS, *n* = 4. ^∗^*p* < 0.05, one-way ANOVA followed by Tukey’s multiple comparison.

Previous studies have suggested that the P2X receptor channel pore dilates following prolonged stimulation (Virginio et al., 1999; Yan et al., 2008) and this raises the possibility that neomycin could be taken up through the ATP-gated ion channels (P2X receptors), accounting for the aggravated hair cell loss induced by ATPγS. Since NTR fluorescence correlates with the concentration of intracellular neomycin (Alharazneh et al., 2011), NTR fluorescence was quantified in the presence of ATPγS to determine if the prolonged activation of P2XR results in additional neomycin uptake. Supplementing the culture medium with ATPγS had no effect on NTR fluorescence in the IHC and OHC of the cochlear apical segment (Figure 4A,B). In the middle segment, NTR fluorescence in IHC was also unaffected by ATPγS supplementation, but in OHC was significantly reduced (from 37.3 ± 2.9 to 22.5 ± 5.4, *p* = 0.017, one-way ANOVA, Figure 4B). In the basal segment, NTR fluorescence was significantly reduced in both IHC (from 22.0 ± 2.7 to 10.7 ± 3.0, *p* = 0.024) and OHC (from 25.2 ± 4.2 to 10.7 ± 4.5, *p* = 0.042) with the addition of ATPγS to the culture medium (*p* < 0.05, one-way ANOVA, Figure 4B). These findings demonstrate that the addition of ATPγS to the culture medium did not increase NTR uptake in the cochlear hair cells, but instead significantly reduced NTR uptake in the basal segment IHC and OHC (Figure 4B). An ATPγS-induced decrease in neomycin uptake in hair cells thus cannot explain the enhancement of neomycin ototoxicity in the presence of a P2 receptor agonist.

### Activation of Adenosine Receptors Mitigates Neomycin-Induced Hair Cell Loss

**Figure 5:**
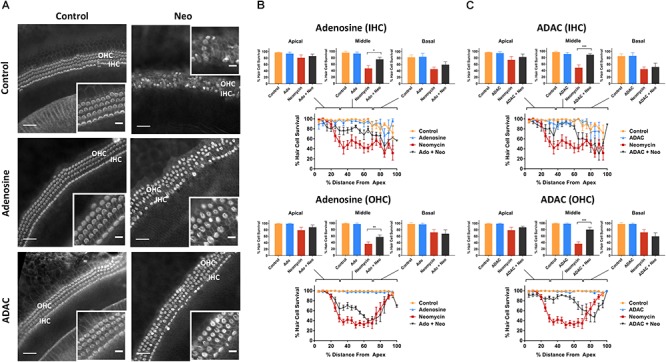
Effect of adenosine or ADAC supplementation on hair cell survival in the neomycin-treated mouse P3 organ of Corti explant. **(A)** Cochlear explants (middle segment) incubated in normal culture medium enriched with adenosine or ADAC for 42 h with or without neomycin administration (1 mM, 3 h) in the middle of the timeframe. IHCs, inner hair cells; OHCs, outer hair cells. Scale bars: main, 30 μm; inset, 10 μm. **(B)** Adenosine (100 μM) supplementation significantly improved the survival of IHC (*p* < 0.05) and OHC (*p* < 0.01, one-way ANOVA) in the middle cochlear turn in the organ of Corti incubated with neomycin. Data presented as mean ± SEM (adenosine, *n* = 5 and adenosine + neomycin, *n* = 6). **(C)** A_1_R agonist ADAC (1 μM) also improved IHC and OHC survival in the middle turn of the cochlea (*p* < 0.001, one-way ANOVA). Data presented as mean ± SEM (ADAC, *n* = 4 and ADAC + neomycin, *n* = 6).

In cochlear explants incubated in culture medium lacking neomycin, supplementation with adenosine (a broadly selective P1 receptor agonist; 100 μM) or ADAC (a preferential adenosine A_1_R agonist; 1 μM) resulted in excellent hair survival rates, similar to the control explants (Figure 5). Moreover, in neomycin-treated cochlear tissues, the organ of Corti retained its organized cytoarchitecture with minimal hair cell loss when adenosine or ADAC were added to the culture medium (Figure 5A). This cytoprotective effect of adenosine and ADAC was restricted to the middle segment of the cochlea where the hair cells are most sensitive to neomycin ototoxicity. Adenosine supplementation significantly increased IHC (from 47.1 ± 8.8 to 75.6 ± 6.0%, *p* = 0.010) and OHC (from 36.0 ± 6.0 to 57.2 ± 6.3%, *p* = 0.0023) survival in the middle cochlear segment (one-way ANOVA; Figure 5B). ADAC supplementation to the culture medium also improved IHC and OHC survival (to 89.4 ± 3.3%, *p* = 0.0003 and 80.2 ± 6.6%, *p* = 0.0000016, respectively) in neomycin-treated tissues in the middle segment of the cochlea (one-way ANOVA; Figure 5C). ADAC, however, conferred a significantly greater otoprotective effect than adenosine. In IHC, cochlear explants incubated with ADAC showed an additional 18% increase in survival rate in comparison to explants incubated with adenosine. Similarly, a 40% increase was observed in OHC survival, suggesting that the protective effect is likely mediated by adenosine A_1_ receptor. In the apical and basal segments, where neomycin did not induce a significant loss of hair cells, adenosine and ADAC supplementation did not affect hair cell survival (Figure 5B,C).

### A_1_R Distribution in the Neonatal (P3) Cochlea

**Figure 6:**
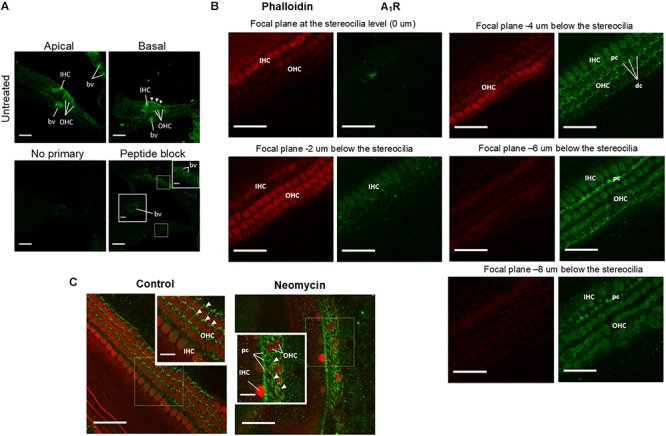
Adenosine A_1_ receptor immunolabeling of the developing mouse organ of Corti explants. **(A)** Cross sections of the P3 mouse cochlea (apical and basal turns) labeled with A_1_R-specific antibody. A_1_R are immunoexpressed in both inner (IHC) and outer (OHC) hair cells. Strong immunolabeling was present in the cuticular plate region of the OHC in the basal turn (arrowheads). Blood vessels (bv) in the basilar membrane were also immunolabeled, but similar labeling was also observed in peptide block controls. The primary antibody was omitted or pre-incubated with the corresponding peptide in control sections. **(B)** Whole mounts of the mouse organ of Corti at P3 showing A_1_R immunoexpression at descending focal planes (2 μm optical sections) starting from the stereocilia level. Images were taken from the middle turn of a control cochlea and double labeled for adenosine A_1_R (green) and phalloidin (red). As expected, phalloidin labeling was restricted to the stereocilia and the cuticular plates of the hair cells. A_1_R immunolabeling was observed in Deiters’ cell (dc) phalangeal processes, cell bodies of IHC, OHC and the pillar cells (pc). **(C)** Whole mounts of the P3 mouse cochlea showing A_1_R immunoexpression (green fluorescence, arrowheads) in the middle segment of the control and neomycin-treated tissues. Neomycin-treated cochlea demonstrates substantial loss of sensory hair cells in the middle turn and enhanced A_1_ immunofluorescence in the supporting (scar) cells (arrowheads). Scale bars: main, 30 μm; inset, 10 μm.

The distribution of A_1_R in the developing (P3) mouse cochlea was investigated using cochlear cryosections and surface preparations of the sensory epithelium. Cross-sections of the developing cochlea were immunolabeled with the adenosine A_1_R antibody (Alomone Labs) and imaged using a laser scanning confocal microscope. A_1_R immunolabeling was predominantly observed in cell bodies of the IHC and OHC, and also on the apical surface (reticular lamina) of the OHC in the basal turn (Figure 6A). Immunofluorescence was absent in cochlear sections incubated without primary antibody (Figure 6A). However, after antigen absorption with the blocking peptide, cochlear cryosections showed weak staining in the endothelial wall of the blood vessels in the basilar membrane and within the stria vascularis (Figure 6A). This suggests that A_1_R labeling in these regions is likely non-specific.

To further analyze A_1_R immunolabeling, wholemount preparations were used to provide surface views of the organ of Corti (Figure 6B). The surface preparations of the organ of Corti confirmed A_1_R immunoexpression in the cell bodies of the IHC and OHC, whilst phalloidin labeling of actin filaments was localized to the actin-rich stereocilia and the cuticular plates of the hair cells (Figure 6B). The A_1_R immunolabeling in the reticular lamina was confined to the phalangeal processes of Deiters’ cells that surround the OHC and the pillar cells between the IHC and the first row of the OHC. Some intracellular A_1_R immunoexpression was observed in the pillar cells (Figure 6B). Neomycin treatment resulted in a substantial loss of sensory hair cells, which were replaced by A_1_R-immunolabeled supporting cells (Figure 6C).

## Discussion

Here, we investigated the role of extracellular nucleotides (ATP, UTP), their analogs (ATPγS, UTPγS) and P1 receptor agonists adenosine and ADAC in the maintenance of sensory hair cells exposed to the ototoxic aminoglycoside, neomycin. Neomycin exposure induced IHC loss in a base-to-apex gradient but a significant OHC resistance to aminoglycoside-induced injury in the extreme basal region of the cochlea was observed. A greater resistance of OHC in the extreme basal end (hook region) of the developing cochlea was unexpected, but was probably a result of the *cadherin 23* mutation responsible for the progressive deafness in C57BL/6 mice (Lin et al., 2018). Mutation in the *cadherin 23* gene can hinder the gating of the mechanotransduction channel and reduce the transduction current and hearing sensitivity (Alagramam et al., 2011). The same mutation can also attenuate aminoglycoside uptake (Vu et al., 2013). It is unclear whether the *Cdh23* mutation is present at P3, but a developmental gradient of *Cdh23* mutation in the extreme basal turn of OHC could explain improved resistance to neomycin in this region (Lin et al., 2018).

Supplementation of culture medium with ATP or UTP in organotypic tissue cultures of the developing (P3) mouse cochlea exposed to neomycin did not affect hair cell survival. This was likely due to the rapid hydrolysis of ATP and UTP by ectonucleotidases expressed in sensory hair cells and supporting cells in the organ of Corti (Vlajkovic et al., 2002) into their respective nucleosides, the process that terminates P2R signaling (Vlajkovic et al., 1998; Zimmermann, 2000). In contrast, the supplementation of slowly hydrolyzable analogs ATPγS and UTPγS, which are resistant to breakdown by ectonucleotidases (Jacobson and Muller, 2016), resulted in segment-specific augmentation of neomycin toxicity. In contrast to the naturally occurring ATP and UTP that are quickly hydrolyzed, prolonged activation of P2 receptors with these analogs could lead to excessive intracellular calcium accumulation which would activate apoptotic pathways and thus aggravate hair cell loss in the neomycin injury model. ATPγS, which can activate both P2X and P2Y receptors (Coddou et al., 2011), reduced the survival of IHC and OHC in the basal region of the neomycin-exposed cochlea, whilst UTPγS, which is a potent and selective agonist at the P2Y_2_/P2Y_4_ receptors (Burnstock, 2014), reduced OHC survival in the middle region of the cochlea. The segment-specific susceptibility of hair cells to ATPγS and UTPγS could be attributed to differential distribution of ATP-gated P2X receptors and UTP-responsive P2Y receptors in the cochlea (Housley et al., 1998; Huang et al., 2010). Although the distribution of P2X and P2Y receptor subunits has been described previously in the neonatal cochlea, the evidence for turn-specific differences in distribution of P2X and P2Y receptors is very limited (Housley et al., 1998; Huang et al., 2010). One study on ATP-activated conductance in isolated OHC demonstrated an increasing conduction gradient from the apical turn to the basal turn (Raybould and Housley, 1997). Although not confirmed by immunohistochemistry, this finding suggests that ATP-gated ion channels (P2X receptors) are predominantly localized in the basal turn of the cochlea (Raybould and Housley, 1997), which could explain the aggravated hair cell loss with ATPγS in this region.

The P2X_2_ ATP-gated ion channels are primarily localized to the endolymph-facing apical pole of the hair cells, including the stereocilia (Housley et al., 1999) and one possible explanation for the P2 receptor-mediated increase in neomycin ototoxicity is enhanced neomycin uptake through the P2X receptor ion channels. Prolonged activation of P2X_2_, P2X_4_, and P2X_7_ subunits can lead to dilation of the receptor pore to accommodate much larger cations (e.g., *N*-methyl-D-glucamine and YO-PRO-1) into the cells (Yan et al., 2008; Volonte et al., 2012). Neomycin has a similar molecule weight as YO-PRO-1 and is also positively charged, raising the possibility of additional neomycin uptake through the enlarged pore of the P2X receptor. We tested this possibility using fluorescently-labeled neomycin (NTR) in the presence of ATPγS but, interestingly, ATPγS appeared to reduce NTR uptake in IHC and OHC, particularly in the basal segment of the cochlea. The NTR study thus does not support P2XR as an alternative route for neomycin entry into the hair cells and cannot directly explain the ATPγS-mediated increase in neomycin ototoxicity. A possible explanation for the reduction in NTR fluorescence may be the impaired MET channel function that limits NTR uptake, or loss of membrane integrity preceding hair cell death, which could lead to the release of intracellular contents including NTR. An alternative explanation for the P2R-mediated increase in ototoxicity is accumulation of [Ca^2+^]_i_ following prolonged stimulation of P2R. In the cochlea and other tissues, activation of P2R leads to an increase in [Ca^2+^]_i_, which is due to calcium influx through the P2XR ion channels, or P2YR-mediated mobilization of [Ca^2+^]_i_ stores (Burnstock, 2014). Indeed, a rise in [Ca^2+^]_i_ has been demonstrated following aminoglycoside exposure and is believed to be integral to aminoglycoside-induced hair cell death (Matsui et al., 2004; Esterberg et al., 2013). P2R activation in the presence of neomycin could further enhance [Ca^2+^]_i_ accumulation and contribute to cell death. Therefore, aggravated hair cell loss induced by ATPγS could be due to calcium dysregulation or increased sensitivity to ATPγS-induced calcium signaling compounding the aminoglycoside-induced cell stress responses.

In contrast to P2 receptor agonists, supplementation of adenosine or ADAC in the culture medium substantially reduced neomycin ototoxicity, particularly in the middle segment of the cochlea, where the hair cells are most sensitive to neomycin. Adenosine activates all subtypes of AR and likely produces a mixed effect on cell survival depending on receptor expression. A_1_, A_2A_, and A_3_ receptors have all been localized to the sensory hair cells, spiral ganglion neurons and the supporting Deiters’ cells in adult Wistar rat cochlea (Vlajkovic et al., 2007). The otoprotective effect of ADAC is predominantly mediated by adenosine A_1_R, based on its pharmacological profile (Jacobson and Gao, 2006). The A_1_R, immunolocalized in IHC and OHC in the juvenile (P3) mouse cochlea (Figure 6A,B), likely mediates the protective effect of ADAC and adenosine on both types of sensory hair cells, consistent with our previous studies (Vlajkovic et al., 2010, 2014, 2017; Wong et al., 2010). Following cochlear injury to the sensory cell, the nearby supporting cells expand, extrude and phagocytose the injured hair cell (Leonova and Raphael, 1997; Bird et al., 2010). This process mediated by ATP (Lahne and Gale, 2008), promotes the removal of injured cells, but is critical in ensuring the integrity of the sensory epithelium and residual hearing preservation.

Changes in the sensory epithelium after neomycin treatment are dynamic with progressive loss of sensory hair cells which are replaced by scar-forming supporting cells. This naturally shifts A_1_R immunoexpression to supporting cells, which was observed in our study (Figure 6C). The A_1_R immunoexpression in these supporting cells may act as a check point in regulating reparative processes and epithelium remodeling. Previous studies have demonstrated significant up-regulation of A_1_R expression in the cochlea after exposure to cisplatin (Ford et al., 1997b) and acoustic stress (Ramkumar et al., 2004) suggesting that the activation of A_1_R is an important cochlear defense mechanism against injury.

Adenosine receptor activation (through adenosine and ADAC) thus confers considerable protection for sensory hair cells exposed to neomycin. Evidence from other studies suggests that this is most likely by reducing oxidative stress and apoptosis via A_1_R activation (Ramkumar et al., 1995, 2015; Vlajkovic et al., 2009). For example, local administration of A_1_R agonist R-PIA onto the round window membrane of the cochlea increases activity of superoxide dismutase (SOD) and glutathione peroxidase, the two principle antioxidant enzymes in the cochlea (Ford et al., 1997a). Previous studies have also demonstrated the efficacy of A_1_R agonists (R-PIA, CCPA, ADAC) as a treatment for cisplatin-induced hearing loss (Whitworth et al., 2004; Gunewardene et al., 2013), by reducing the loss of sensory hair cells, expression of oxidative stress markers and apoptosis in the organ of Corti and stria vascularis after cisplatin administration. Adenosine A_1_ receptor activation also protects against cisplatin ototoxicity by suppressing the NOX3/STAT1 inflammatory pathway in the cochlea (Kaur et al., 2016). In addition, stimulation of A_1_R inhibits voltage-gated Ca^2+^ channels, and reduced Ca^2+^ influx can prevent the activation of apoptotic and necrotic cell death pathways (Vlajkovic et al., 2009; Wong and Ryan, 2015). In contrast, A_2A_R activation aggravates ototoxic injury (Whitworth et al., 2004).

Based on this experimental evidence, we suggest that extracellular nucleosides and nucleotides differentially regulate the response of the cochlea to injury, in this case from neomycin. Whilst the prolonged activation of P2 receptor is shown to be detrimental to sensory hair cells, others have suggested that the immediate increase of ATP in the extracellular space following injury may be necessary for the initiation of the reparative processes in the inner ear (Lahne and Gale, 2008; Kuipers et al., 2014). The presence of ATP-hydrolyzing enzymes (ectonucleotidases) in the cochlear tissue (Vlajkovic et al., 2002) may act as a regulatory mechanism preventing excessive P2 receptor stimulation in sensory hair cells by shifting the action from P2 receptor to P1 receptor signaling. The protective effect of adenosine and ADAC is most likely due to A_1_R-mediated increase in antioxidant responses, which has been demonstrated in various cochlear injury models including noise- and drug-induced hearing loss (Hu et al., 1997; Whitworth et al., 2004; Vlajkovic et al., 2010; Gunewardene et al., 2013). A_1_R are strategically distributed adjacent to the P2 receptors in the organ of Corti, and could act as a protective mechanism by increasing endogenous antioxidant defenses and promoting sensory hair cell survival.

## Conclusion

In this study, we investigated the role of purinergic P1 (adenosine) and P2 (ATP, UTP) receptors on hair cell survival in cochlear explants exposed to ototoxic aminoglycoside antibiotic neomycin. Our study shows that supplementing the culture medium with P1 and P2 receptor agonists differentially affects hair cell survival. While prolonged activation of P2 receptors is detrimental to cochlear hair cell survival, the activation of P1 receptors (probably the A_1_ subtype) confers hair cell protection against neomycin ototoxicity in organotypic tissue cultures of the developing mouse cochlea. This suggests that purinergic signaling modulates the balance of pro-survival and pro-death pathways in hair cells of the cochlea exposed to ototoxic drugs.

## Ethics Statement

This study was carried out in accordance with the recommendations of The University of Auckland Animal Ethics Committee. The protocols AEC871 and AEC1339 were approved by The University of Auckland Animal Ethics Committee.

## Author Contributions

SL performed the experiments with SV and PT overseeing experiments and data analysis. All authors were involved in designing and planning of the study and contributed to the writing and editing of the manuscript.

## Conflict of Interest Statement

The authors declare that the research was conducted in the absence of any commercial or financial relationships that could be construed as a potential conflict of interest.
